# On the Cold Water Treatment of Preissnitz at Graeffenberg

**Published:** 1842-06-04

**Authors:** 


### ANALECTA.

### *A Letter on the Cold Water Treatment of Preissnitz at Graeffenberg.*

[We have already once or twice called the attention of our readers to the new folly of the day, Hydrosudopathy. The following very graphic description comes from a patient—we should say a martyr to the process.]

"It requires great patience and perseverance to go through the cold water cure, and it is decidedly the most discouraging of any other. I shall now give you an exact account of what I have to go through every day, as well as all other persons who are able to bear it. The attendant comes into my room at 5 o'clock, A. M. with a wet sheet as cold as ice. I get up, while he puts a large thick blanket on the bed over which he lays the sheet, which I have to stretch myself on, when he wraps it close all about me, and then regularly packs me up, so that I am unable to stir a limb, and then puts a soft down bed over all. I remain in that enviable state for one hour; my first impression was that I should be perished to death, but in about fifteen minutes I was in a most comfortable heat and generally slept until 6 o'clock, when the man enters and unpacks me, taking the sheet away leaving the blanket close about me, when I then go to the bath room, which is situated but a few steps from my bed room, when I plunge in head and heels. I am then well dried, and after being dressed, I set out to walk until 8 o'clock, when I go to breakfast, first taking three or four tumblers of cold water, and about six more before night. Fancy about three hundred persons from all nations sitting down to breakfast on bread, butter, and milk, there being no tea or coffee allowed; we have also abundance of strawberries, so that I assure you we all feel perfectly content, and enjoy it much: at 10 o'clock I must be in my room to undress and take a douche bath, which at first I did not get enamoured with. The water is conveyed in wooden pipes from the mountain, and is as cold as possible. When that is over and I am dressed,

I have but a short time to walk, as I must be in my room again at half past 11 o'clock, where a sitz bath is brought in, where I remain sitting for twenty minutes, after which I have until 1 o'clock to amuse myself in the best way I can, when the dinner bell rings and we all congregate as we did in the morning. The room is a very fine one, being 120 feet long by 50 broad and capable of dining 500 persons, which number and more are here under cure, but a great many stop at Frywalden, a small and neat town one mile, and where Prissnitz goes twice a day to visit his patients. You would suppose I had the remainder of the day to myself, but not so, for at 5 o'clock I must again go into the wet sheet, and take the bath after, as in the morning. We meet at 8 to supper, which consists of the same fare as at breakfast; I am generally in bed by ten, but not before the man comes in with two long wet linen bandages, which he puts round my waist, and at the back of the neck, and between my shoulders, by way of making me *comfortable* for the night. You can fancy from the above how I am to pass my days here, that I have not much idle time on my hands. I did not mention that in addition, I have to swallow from twelve to fifteen tumblers of cold water. Graffenberg is a very high hill in the centre of three valleys, and what makes it very picturesque, it is all finely wooded at the top, and walks made all through, and on the sides, with numerous seats of all dimensions, placed for the company: and an amateur band comes from Frywalden twice a week to play, and on every Sunday there is a full band that plays all the time we are at dinner, and again after supper, when such as are inclined, commence dancing."—*Dublin Jour. Med. Sci.* May, 1842.

